# Gamma-glutamyl transferase to high-density lipoprotein cholesterol ratio: A valuable predictor of type 2 diabetes mellitus incidence

**DOI:** 10.3389/fendo.2022.1026791

**Published:** 2022-09-29

**Authors:** Wangcheng Xie, Bin Liu, Yansong Tang, Tingsong Yang, Zhenshun Song

**Affiliations:** ^1^ Department of General Surgery, Shanghai Tenth People’s Hospital, Tongji University School of Medicine, Shanghai, China; ^2^ Department of Gastrointestinal Surgery, Anqing First People’s Hospital, Anhui Medical University, Anqing, China; ^3^ Department of Cardiology, Shanghai Tenth People’s Hospital, Tongji University School of Medicine, Shanghai, China

**Keywords:** gamma-glutamyl transferase, high-density lipoprotein cholesterol, incidence, risk, type 2 diabetes mellitus

## Abstract

**Background:**

Gamma-glutamyl transferase (GGT) and high-density lipoprotein cholesterol (HDL-C) have been proven to be valuable predictors of type 2 diabetes mellitus (T2DM). The aim of this study was to investigate the association between GGT/HDL-C ratio and incident T2DM.

**Methods:**

The study retrospectively analyzed 15453 participants from 2004 to 2015. Cox proportional hazards regression models and Kaplan-Meier curves were used to elucidate the effect of GGT/HDL-C ratio on T2DM. Restricted cubic spline (RCS) analysis was performed to explore any non-linear correlation between GGT/HDL-C ratio and the risk of T2DM. The predictive performance of GGT, HDL-C and GGT/HDL-C ratio for T2DM was evaluated utilizing receiver-operating-characteristic (ROC) curves.

**Results:**

During a median follow-up of 5.39 years, 373 cases of incident T2DM were observed. Kaplan-Meier curves showed that the cumulative probabilities of T2DM increased in the participants with higher GGT/HDL-C ratio significantly (P < 0.001). Cox models further clarified that high GGT/HDL-C ratio was an independent risk factor for T2DM (HR = 1.01, 95% CI = 1.00-1.01, P = 0.011). Linear positive correlation between GGT/HDL-C ratio and the risk of T2DM was demonstrated through RCS analysis. In the ROC analysis, GGT/HDL-C ratio (AUC = 0.75, 95% CI = 0.73-0.77) showed competitive role in the prediction of T2DM compared with single GGT and HDL-C.

**Conclusions:**

The GGT/HDL-C ratio could serve as a valuable predictor of T2DM, and the risk of T2DM increases in the condition of higher GGT/HDL-C ratio.

## Introduction

With the improvement of living standards, diabetes mellitus has become an increasingly common disease, causing a serious public burden (1). According to the latest statistics from the International Diabetes Federation, there are at least 537 million adult living with diabetes mellitus worldwide now, and it is expected to reach a terrifying 783 million by 2045 (2). Diabetes mellitus can be mainly divided into type 1 diabetes mellitus (T1DM) and type 2 diabetes mellitus (T2DM), with the latter accounting for over 90% (3). Given that T2DM is a preventable disease, early management of high risk patients was restricted to the lack of efficient forecasting tools.

The pathogenesis of T2DM is complex, with insulin resistance and β-cell dysfunction being the most important (3). Liver is one of the major target organs of insulin. Several studies have pointed out that liver dysfunction is closely related to the development of T2DM (4, 5). Gamma-glutamyl transferase (GGT), a common marker of liver dysfunction, has been shown to be independently associated with insulin resistance (6). Further studies have demonstrated that GGT could be an important predictor of T2DM and that the risk of T2DM increases with elevated GGT (7, 8).

On the other hand, metabolic syndrome is recognized as an important risk factor for T2DM and is characterized by the decline of high-density lipoprotein cholesterol (HDL-C) concentrations (3). HDL-C has the ability to stimulate glucose uptake and improve β-cell function, and its decrease can reflect reduced β-cell function and insulin resistance (9). To date, multiple works have established that decreased HDL-C levels are associated with an increased risk of T2DM (9, 10).

GGT/HDL-C ratio was a new indicator proposed by a recent study (11).The study found that GGT/HDL-C ratio could serve as a predictor of non-alcoholic fatty liver disease (NAFLD) prevalence and was more efficient than single GGT or HDL-C significantly. However, there is still a lack of studies reporting the association of GGT/HDL-C ratio with other diseases. Therefore, the aim of this study was to evaluate the predictive performance of GGT/HDL-C ratio for T2DM compared with single GGT and HDL-C.

## Materials and methods

### Participants

**Figure 1:**
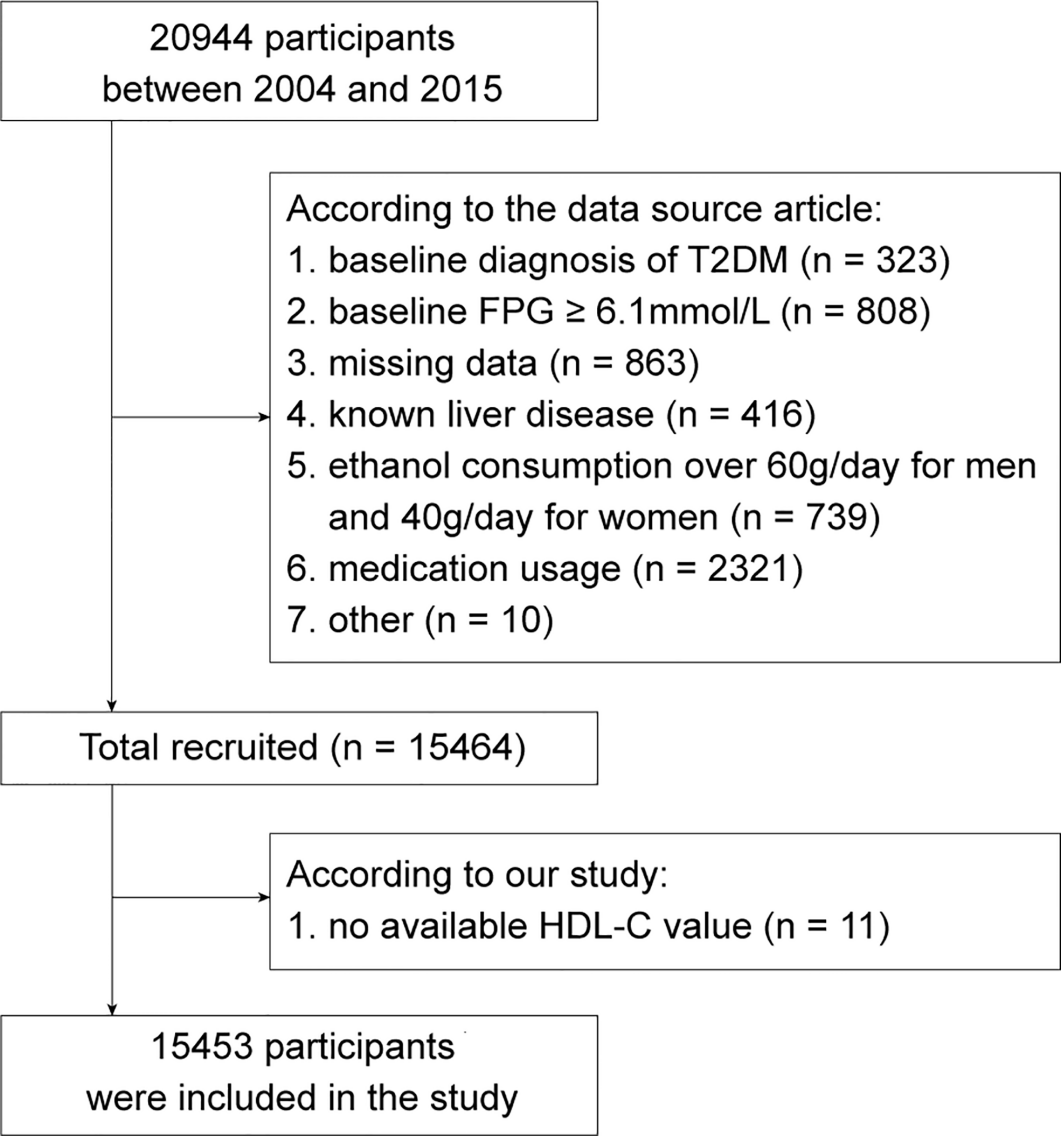
The flow chart of study participants selection. FPG, fasting plasma glucose; HDL-C, high-density lipoprotein cholesterol. T2DM, type 2 diabetes mellitus.

The original data for this study were obtained from a publicly available dataset within the DRYAD database (https://doi.org/10.5061/dryad.8q0p192) (12). The dataset was built by Okamur et al. based on a medical examination program conducted at Murakami Memorial Hospital (Gifu, Japan) from 2004 to 2015. This retrospective cohort initially recruited 20944 participants who completed at least 2 scheduled examinations within a specified time frame. According to the study design of Okamur et al, participants who met the following criteria had been excluded: 1. baseline diagnosis of T2DM (n = 323); 2. baseline fasting plasma glucose (FPG) ≥ 6.1mmol/L (n = 808); 3. missing data of covariates (n = 863); 4. known liver disease, such as alcoholic fatty liver disease and viral hepatitis (n = 416); 5. ethanol consumption over 60g/day for men and 40g/day for women (n = 739); 6. medication usage (n = 2321) (12). In addition, 10 participants were excluded by the researchers for unknown reasons. Thus, the public dataset obtained contained a total of 15464 participants. Next, 11 participants with missing HDL-C value were further eliminated in accordance with the aim of this study. Finally, there were 15453 participants enrolled in the study cohort. (**Figure 1**)

The study of Okamur et al. was approved by the Murakami Memorial Hospital Ethics Committee and all participants signed an informed consent form (12). This study complied with the public policy statement of the DRYAD database and was therefore obtained an ethical exemption according to the Ethics Committee of Shanghai Tenth People’s Hospital.

### Data collection and definition

All participants completed a standardized self-administration questionnaire to provide medical history and lifestyle factors including alcohol consumption, smoking status, and habit of exercise. Additionally, body mass index (BMI), systolic blood pressure (SBP) and diastolic blood pressure (DBP) were obtained by physical examination, as well as FPG, glycated hemoglobin (HbA1c), alanine aminotransferase (ALT), aspartate transaminase (AST), GGT, HDL-C, cholesterol and triglycerides by analyzing fasting blood samples.

Meanwhile, Okamur et al. provided clear definitions for the following indicators (13). Alcohol consumption was estimated based on the type and amount of alcohol consumed by participants in the previous month per week and was categorized into 4 levels: no or minimal (<40g/week), light (40-140g/week), moderate (140-280g/week) and heavy (>280g/week). Habit of exercise was defined as regular exercise in any type more than once a week. Furthermore, fatty liver was diagnosed by experienced technologists and gastroenterologists by abdominal ultrasound. Finally, incident T2DM was defined as HbA1c ≥ 6.5%, FPG ≥ 7 mmol/L or self-reported.

### Statistical analysis

Continuous variables would be described as mean ± standard deviation if they follow a normal distribution and compared using the Student’s t-test. Otherwise they would be reported as median (quartiles) and compared utilizing the Mann-Whitney U test. Categorical variables were expressed as frequency (percentage) and evaluated with chi-square test. Then, GGT/HDL-C ratio was divided into 4 groups based on quartiles, namely Q1 (<7.20), Q2 (7.20-10.78), Q3 (10.78-17.96) and Q4 (>17.96). Kaplan-Meier curves were applied to visualize the cumulative probability of T2DM in each group. Next, four Cox proportional hazards regression models were developed to investigate the independent effect of GGT/HDL-C ratio on T2DM, among which three adjusted models contributed to reducing bias. Model 1 was adjusted for age and gender. Model 2 was further adjusted for BMI, alcohol consumption, smoking status and fatty liver on the basis of model 1. Model 3 was further adjusted for habit of exercise, FPG, HbA1c, ALT, AST, cholesterol, triglycerides, SBP and DBP on the basis of model 2. Based on model 3, the restricted cubic spline (RCS) model with 4 knots was employed to explore any non-linear correlation between GGT/HDL-C ratio and the risk of T2DM. Finally, in order to assess the efficiency of GGT/HDL-C ratio in predicting T2DM, receiver-operating-characteristic (ROC) curves were performed and the best cut-off value were further obtained.

All statistics and analyses were performed by SPSS (version 26.0) and R software (version 4.0.5). P values (two-sided) below 0.05 were considered to indicate statistical significance.

## Results

A total of 15453 participants were recruited into the final retrospective cohort study. 373 of them (2.41%) developed incident T2DM after a median follow-up period of 5.39 years. It was clear that participants with T2DM were older and higher in the percentage of male than those without T2DM. Meanwhile, T2DM group involved in more participants with characteristics of heavy alcohol consumption, current smoking, fatty liver and no exercise habit. In addition, the baseline BMI, FPG, HbA1c, ALT, AST, GGT, cholesterol, triglycerides, SBP and DBP were higher but HDL-C was lower in T2DM group. The GGT/HDL-C ratio was further calculated. Its median was only 10.62 in non-T2DM group, whereas it reached 21.53 in T2DM group, showing a dramatically significance (P < 0.001). (**Table 1**)

**Table 1:** Baseline characteristics of the study participants.

Variables	Total (n = 15453)	T2DM (n = 373)	Non-T2DM (n = 15080)	P value
Follow-up duration, years	5.39 (2.70, 9.38)	5.93 (2.97, 8.92)	5.36 (2.69, 9.44)	0.670
Age, years	43.71 ± 8.90	47.14 ± 8.52	43.63 ± 8.89	< 0.001
Gender, n (%)				< 0.001
^ ^Male	8419 (54.48)	286 (76.68)	8133 (53.93)	
^ ^Female	7034 (45.52)	87 (23.32)	6947 (46.07)	
BMI, kg/m^2 ^	22.12 ± 3.13	25.03 ± 3.82	22.04 ± 3.07	< 0.001
Alcohol consumption, n (%)				< 0.001
^ ^No or minimal	11802 (76.37)	266 (71.31)	11536 (76.50)	
^ ^Light	1754 (11.35)	40 (10.72)	1714 (11.37)	
^ ^Moderate	1357 (8.78)	37 (9.92)	1320 (8.75)	
^ ^Heavy	540 (3.49)	30 (8.04)	510 (3.38)	
Smoking status, n (%)				< 0.001
^ ^Never	9027 (58.42)	145 (38.87)	8882 (58.90)	
^ ^Past	2949 (19.08)	77 (20.64)	2872 (19.05)	
^ ^Current	3477 (22.50)	151 (40.48)	3326 (22.06)	
Fatty liver, n (%)				< 0.001
^ ^No	12716 (82.29)	150 (40.21)	12566 (83.33)	
^ ^Yes	2737 (17.71)	223 (59.79)	2514 (16.67)	
Habit of exercise, n (%)				0.048
^ ^No	12747 (82.49)	322 (86.33)	12425 (82.39)	
^ ^Yes	2706 (17.51)	51 (13.67)	2655 (17.61)	
FPG, mmol/L	5.16 (4.88, 5.44)	5.72 (5.38, 5.88)	5.16 (4.88, 5.44)	< 0.001
HbA1c, %	5.17 ± 0.32	5.53 ± 0.37	5.16 ± 0.32	< 0.001
ALT, IU/L	17.00 (13.00, 23.00)	24.00 (18.00, 39.00)	17.00 (13.00, 23.00)	< 0.001
AST, IU/L	17.00 (14.00, 21.00)	20.00 (16.00, 26.00)	17.00 (14.00, 21.00)	< 0.001
GGT, IU/L	15.00 (11.00, 22.00)	24.00 (16.50, 36.50)	15.00 (11.00, 22.00)	< 0.001
HDL-C, mmol/L	1.46 ± 0.40	1.19 ± 0.33	1.47 ± 0.40	< 0.001
Cholesterol, mmol/L	5.13 ± 0.86	5.43 ± 0.90	5.12 ± 0.86	< 0.001
Triglycerides, mmol/L	0.73 (0.50, 1.12)	1.21 (0.86, 1.93)	0.72 (0.49, 1.11)	< 0.001
SBP, mmHg	114.49 ± 14.97	122.03 ± 15.59	114.31 ± 14.91	< 0.001
DBP, mmHg	71.58 ± 10.50	77.18 ± 10.23	71.44 ± 10.47	< 0.001
GGT/HDL-C	10.78 (7.20, 17.96)	21.53 (13.12, 34.23)	10.62 (7.15, 17.58)	< 0.001
^ ^P for trend				< 0.001
^ ^Q1	3863	20 (5.36)	3843 (25.48)	
^ ^Q2	3863	42 (11.26)	3821 (25.34)	
^ ^Q3	3863	75 (20.11)	3788 (25.12)	
^ ^Q4	3864	236 (63.27)	3628 (24.06)	

ALT, alanine aminotransferase; AST, aspartate transaminase; BMI, body mass index; DBP, diastolic blood pressure; FPG, fasting plasma glucose; GGT, gamma-glutamyl transferase; HbA1c, glycated hemoglobin; HDL-C, high-density lipoprotein cholesterol; SBP, systolic blood pressure; T2DM, type 2 diabetes mellitus.

**Figure 2:**
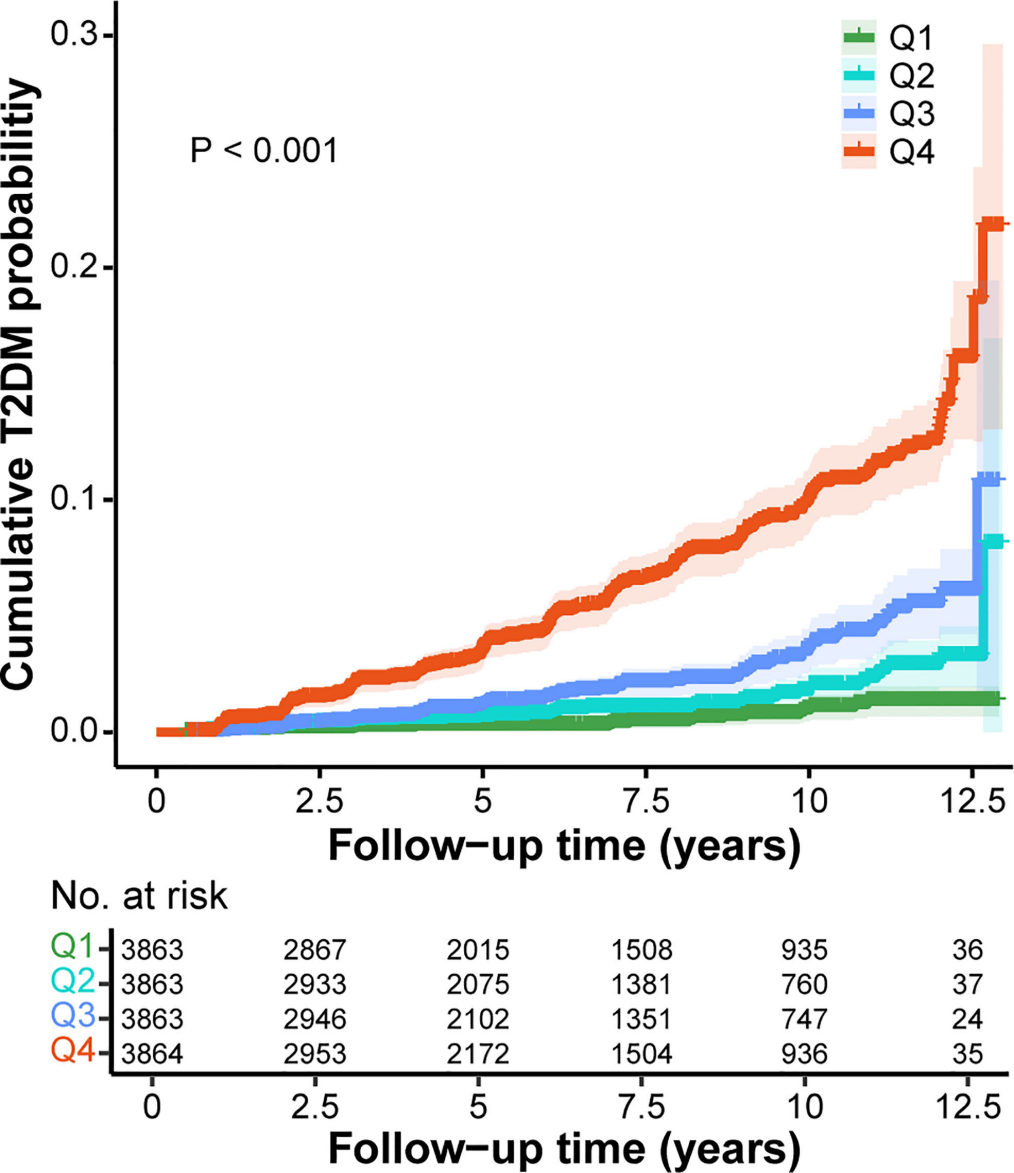
Kaplan-Meier curves for the cumulative T2DM probability according to different GGT/HDL-C ratio. Q1, < 7.20; Q2, 7.20-10.78; Q3, 10.78-17.96; Q4, >17.96. GGT, gamma-glutamyl transferase; HDL-C, high-density lipoprotein cholesterol; T2DM, type 2 diabetes mellitus.

Subsequently, GGT/HDL-C ratio was divided into four groups based on quartiles. It was apparent that the distribution of GGT/HDL-C ratio in non-T2DM group was balanced, with each group accounting for approximately 25%. However, 63.27% of participants with T2DM were in the highest Q4 group, while only 5.36% were in Q1 group. (**Table 1**) Kaplan-Meier analysis was further used to explore the effect of different levels of GGT/HDL-C ratio on the cumulative probability of T2DM. Evidently, the cumulative T2DM probabilities increased in the condition of higher GGT/HDL-C ratio (P < 0.001). (**Figure 2**)

Next, univariate and multivariate Cox proportional hazards regression models were constructed to investigate the independent effect of GGT/HDL-C ratio on incident T2DM. In the univariate Cox model, elevated GGT/HDL-C ratio would increase the risk of T2DM (HR = 1.02, 95% CI = 1.01-1.02, P < 0.001). Similarly, the risk of T2DM was significant higher in groups Q2, Q3 and Q4 with higher GGT/HDL-C ratios compared to Q1 group, with Q4 group being 11.60 times higher than Q1 group (HR = 11.60, 95% CI = 7.35-18.31, P < 0.001). Model 1 and model 2, which were further adjusted for some covariates, showed similar results. The final model 3 adjusting for all covariates illustrated that GGT/HDL-C ratio was still an independent risk factor for T2DM as a continuous variable (HR = 1.01, 95% CI = 1.00-1.01, p = 0.011). Interestingly, when GGT/HDL-C ratio was considered as a categorical variable, no statistical difference was shown between Q2 and Q3 groups and Q1 group, but the risk of T2DM in Q4 group still was demonstrated to be 1.97 times higher than Q1 group (HR = 1.97, 95% CI = 1.13-3.44, P = 0.018). (**Table 2**)

**Table 2:** Cox proportional hazards regression analysis for the association between GGT/HDL-C ratio and incident T2DM.

Variables	Univariate analysis		Multivariate analysis					
			Model 1		Model 2		Model 3	
	HR (95% CI)	P value	HR (95% CI)	P value	HR (95% CI)	P value	HR (95% CI)	P value
GGT/HDL-C	1.02 (1.01, 1.02)	< 0.001	1.01 (1.01, 1.02)	< 0.001	1.01 (1.00, 1.01)	< 0.001	1.01 (1.00, 1.01)	0.011
P for trend		< 0.001		< 0.001		< 0.001		0.007
^ ^Q1	Ref		Ref		Ref		Ref	
^ ^Q2	2.22 (1.30, 3.78)	0.003	2.13 (1.25, 3.63)	0.005	1.80 (1.05, 3.09)	0.034	1.29 (0.75, 2.23)	0.356
^ ^Q3	4.04 (2.47, 6.62)	< 0.001	3.72 (2.27, 6.10)	< 0.001	2.28 (1.33, 3.89)	0.003	1.27 (0.74, 2.18)	0.392
^ ^Q4	11.60 (7.35, 18.31)	< 0.001	10.49 (6.64, 16.58)	< 0.001	4.30 (2.50, 7.37)	< 0.001	1.97 (1.13, 3.44)	0.018

Model 1 was adjusted for age and gender. Model 2 was further adjusted for BMI, alcohol consumption, smoking status and fatty liver on the basis of model 1. Model 3 was further adjusted for habit of exercise, FPG, HbA1c, ALT, AST, cholesterol, triglycerides, SBP and DBP on the basis of model 2. ALT, alanine aminotransferase; AST, aspartate transaminase; BMI, body mass index; CI, confidence interval; DBP, diastolic blood pressure; FPG, fasting plasma glucose; GGT, gamma-glutamyl transferase; HbA1c, glycated hemoglobin; HDL-C, high-density lipoprotein cholesterol; HR, hazard ratio; SBP, systolic blood pressure; T2DM, type 2 diabetes mellitus.

**Figure 3:**
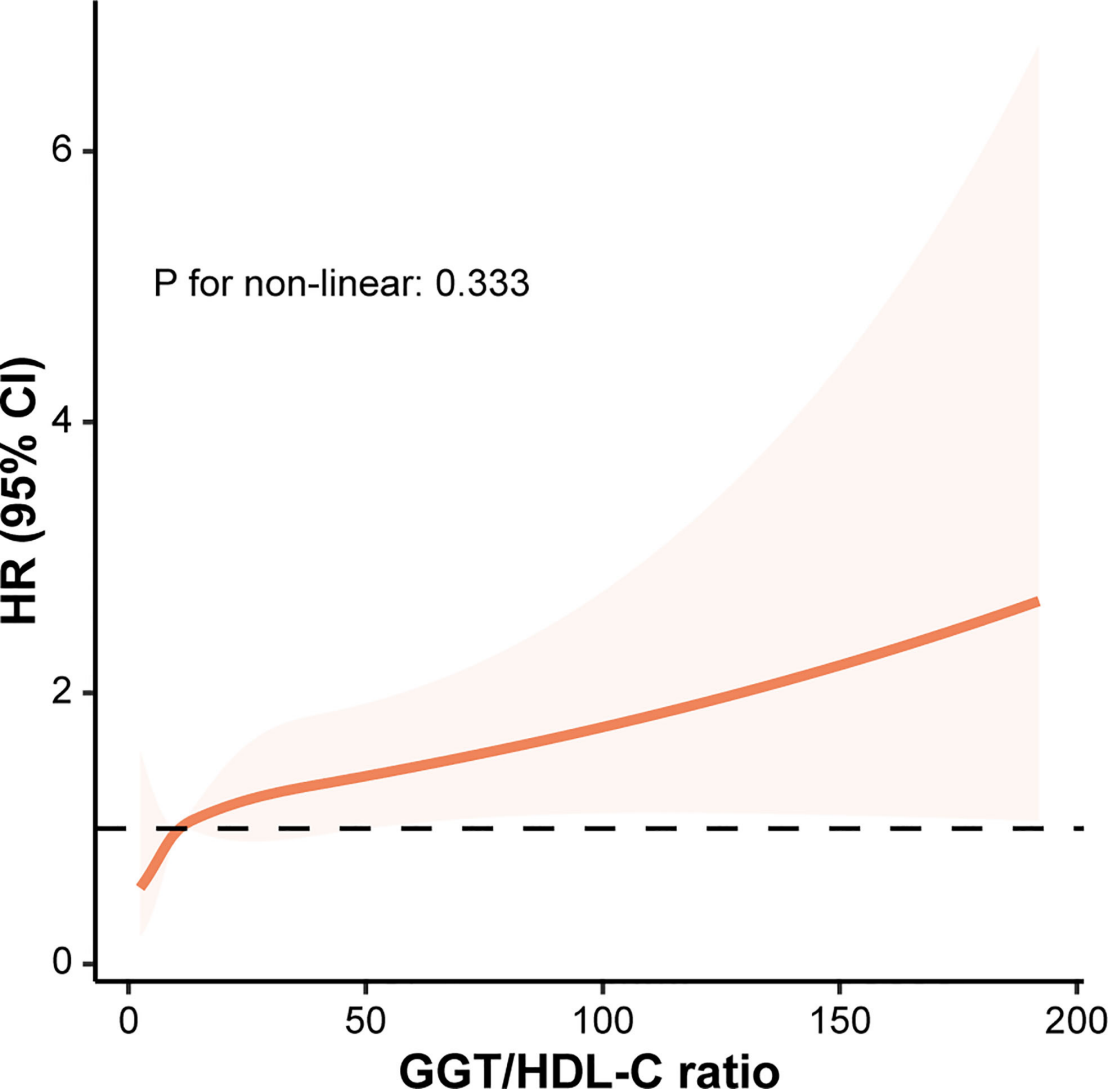
The correlation between GGT/HDL-C ratio and the risk of T2DM evaluated by restricted cubic splines. CI, confidence interval; GGT, gamma-glutamyl transferase; HDL-C, high-density lipoprotein cholesterol; HR, hazard ratio; T2DM, type 2 diabetes mellitus.

RCS model was established based on model 3 to examine whether there was a non-linear correlation between GGT/HDL-C ratio and the risk of T2DM. Obviously, GGT/HDL-C ratio was linearly correlated with the risk of T2DM due to a P for non-linear of 0.333. And the risk of T2DM increased with elevated GGT/HDL-C ratio. (**Figure 3**)

**Figure 4:**
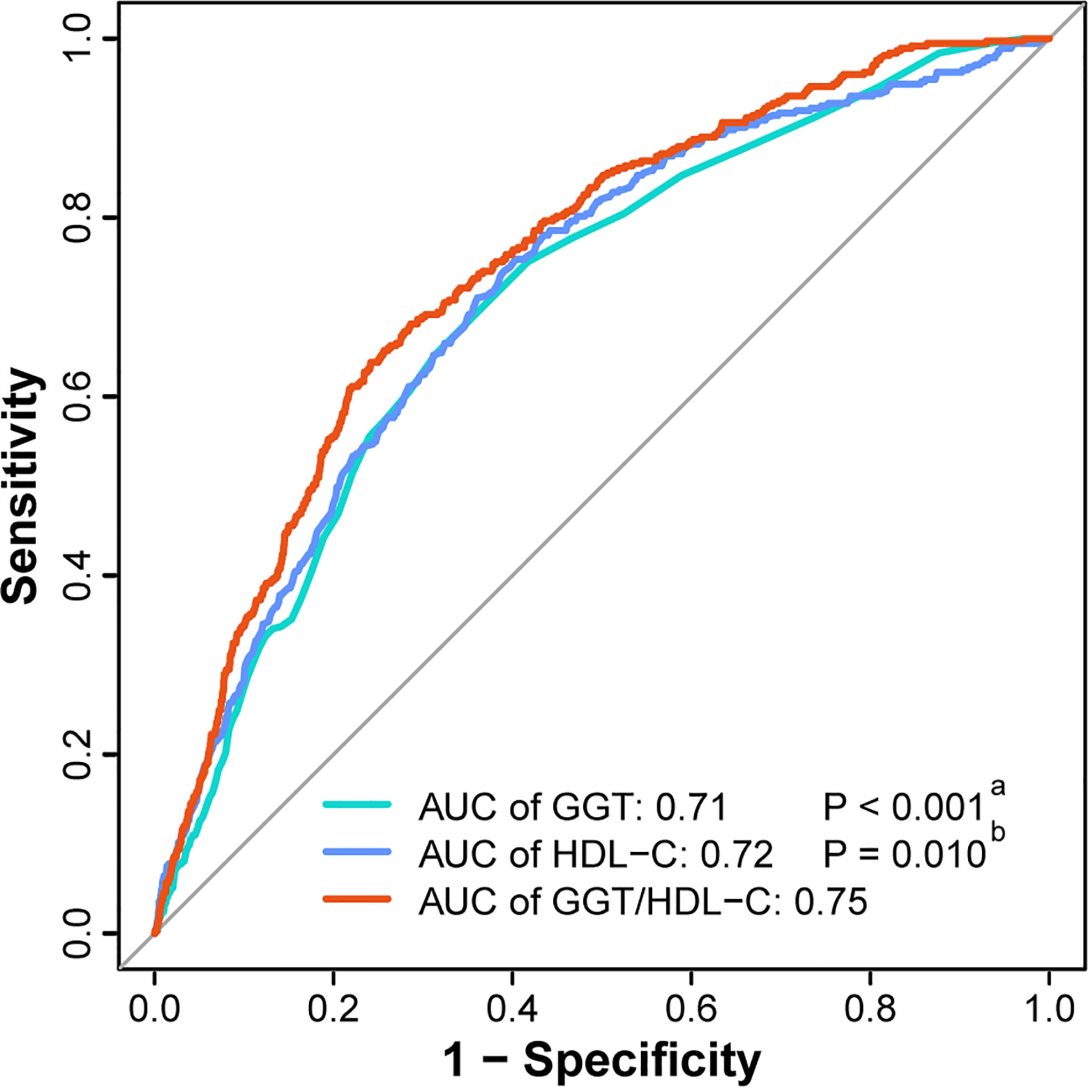
ROC curves of GGT, HDL-C and GGT/HDL-C ratio to predict incident T2DM. **(^a^)** The P value of GGT compared with GGT/HDL-C ratio. **(^b^)** The P value of HDL-C compared with GGT/HDL-C ratio. AUC, area under the curve; GGT, gamma-glutamyl transferase; HDL-C, high-density lipoprotein cholesterol; ROC curve, receiver-operating-characteristic curve; T2DM, type 2 diabetes mellitus.

Finally, ROC analysis was utilized to elucidate the predictive performance of GGT/HDL-C ratio in incident T2DM. Apparently, GGT/HDL-C ratio (AUC = 0.75, 95% CI = 0.73-0.77) was a better predictor when compared with single GGT (AUC = 0.71, 95% CI = 0.68-0.73, P < 0.001) or single HDL-C (AUC = 0.72, 95% CI = 0.69-0.74, P = 0.010). (**Figure 4**) In addition, the best cut-off value for GGT/HDL-C ratio was 17.92 (sensitivity of 0.638, specificity of 0.758).

## Discussion

In brief, this was the first study to explore the association between GGT/HDL-C ratio and the risk of T2DM. Furthermore, the large population-based retrospective cohort study demonstrated that GGT/HDL-C ratio was linearly correlated with the risk of T2DM and that the risk of T2DM increased in the condition of higher GGT/HDL-C ratio. GGT/HDL-C ratio could serve as a valuable predictor of T2DM with better predictive performance compared to single GGT and HDL-C.

T2DM and its complications have become a serious threat to the quality of life of patients. Recent studies on the epidemiology of T2DM have revealed numerous risk factors, such as genetics, obesity, metabolic syndrome, unhealthy diet, unhealthy lifestyle habits, smoking and alcohol consumption, and these factors interact in the development and progression of T2DM (3). Given the preventable character of T2DM and the high clinical benefits of early diagnosis and treatment, it is essential and important to screen high-risk individuals through simple and practical indicators (14). Currently, researchers have developed a variety of novel predictive markers, including triglycerides, phospholipids, aromatic amino acids, branched-chain amino acids, hexoses and more (15).

Recently, numerous researchers have suggested that liver enzymes could be potential predictors of the incidence of diabetes (16, 17). Liver enzymes are widely used in clinical practice to evaluate liver function and have the advantage of being economical and easy to test. It is well known that liver dysfunction is strongly associated with the prevalence of T2DM (5). On the one hand, liver plays a key role in the regulation of glucose homeostasis, as insulin can effectively stimulates hepatic glycogen synthesis and reduces hepatic glucose production (4, 18). On the other hand, insulin has the ability to directly affect lipid metabolism in hepatocytes, for example by up-regulating genes in the *de novo* lipogenesis (DNL) pathway and decreasing the export of very low density lipoproteins (4, 19). Intriguingly, human insulin resistance is highly correlated with hepatic steatosis, a phenomenon known as “selective hepatic insulin resistance” (20, 21). Furthermore, the interaction between insulin resistance and NAFLD is reciprocal. Insulin resistance promotes intrahepatic lipid accumulation and liver damage, while lipid accumulation and cytokine release in turn promote intrahepatic insulin resistance (22). Therefore, as common markers of liver function, blood ALT, AST and GGT levels have all been shown to correlate with insulin resistance and the risk of T2DM, among which GGT displays the strongest correlation (16, 17, 23). In recent years, several large cohort studies demonstrated the excellent predictive performance of GGT for T2DM (24, 25). And a Mendelian randomization study provided genetic evidence for a causal role of GGT in the development of T2DM (8). In addition to being able to indirectly reflect insulin resistance by representing liver dysfunction and intrahepatic lipid accumulation, GGT is also present in other tissues and is involved in oxidative stress through the regulation of glutathione, which is associated with the pathophysiological processes of diabetes (26, 27). It has been suggested that elevated GGT values indicate subclinical inflammation in the body, which also contributes to the development of T2DM (28). Not only is GGT positively associated with a number of inflammatory agents, such as C-reactive protein and interleukin-6, but itself also mediates the conversion of leukotriene C4 to leukotriene D4, which enhances the development of inflammation (25, 29).

Traditionally, HDL-C has often been considered to be strongly associated with cardiovascular events (30). However, several studies pointed to the association of lower HDL-C with higher incidence of T2DM in recent years, suggesting that HDL-C could be a valuable predictor of T2DM (31, 32). Meanwhile, a wide-angled mendelian randomization study elucidated that HDL-C was a protective factor for T2DM (33). HDL-C has the capacity to regulate skeletal muscle glucose uptake and β-cell insulin secretion, since some researchers discovered that intravenous HDL-C significantly reduced plasma glucose concentrations (32, 34, 35). At the same time, HDL particles act as major receptors of cholesterol and contribute to the protection of β-cells from apoptosis due to cholesterol accumulation by promoting cholesterol efflux (9). HDL-C also has antioxidant and anti-inflammatory properties that directly increase β-cell survival through inhibition of apoptosis caused by oxidized low density lipoprotein and inflammatory factors (10). Besides this, HDL-C can reflect genomic changes, for example, changes in *HMGCR* polymorphisms can lead to a reduction in HDL-C, which is thought to be associated with the risk of T2DM (36). *ABCA1* gene variants are capable of contributing to T2MD development by reducing HDL-C levels leading to β-cell lipotoxicity (37). In addition to concentration, the particle size of HDL has been proved to be associated with the maintenance of glucose homeostasis. Large spherical HDL was negatively associated with worsening insulin resistance, while smaller TAG-rich HDL particles were positive associated with elevated blood glucose (38, 39).

Given that most researchers currently hypothesized that GGT is positively and HDL-C is negatively correlated with T2DM, it is suspected that the combined GGT/HDL-C ratio might possess better predictive performance compared with the individual metrics. The results of the ROC analysis in this study validated the assumption well for the first time. A recent research reported a similar result that explored the predictive performance of GGT/HDL-C ratio for the risk of NAFLD (11).They found the AUC of GGT/HDL-C ratio was 0.799, which was higher than single GGT (AUC = 0.773) and single HDL-C (AUC = 0.726) significantly. In addition, the best cut-off value was 21.3, slightly higher than in this study. It is not difficult to understand the better predictive performance of GGT/HDL-C ratio for NAFLD compared with T2DM, as GGT and HDL-C have a stronger association with NAFLD. Regardless, GGT and HDL-C are common in clinical testing for non-diabetic patients, so the GGT/HDL-C ratio is indeed a convenient and cost-effective index to screen for T2DM. At the same time, the best cut-off value for the GGT/HDL-C ratio was 17.92, which means it is important to focus on populations whose GGT/HDL-C ratio is over 17.92, because they are at high risk of T2DM. Guidance from clinical practitioners on improving their lifestyle and promoting healthy diet and physical activity is essential to help them achieve early prevention or delay of T2DM.

There were also some potential limitations to this study. Firstly, this was a retrospective study. The principal drawback was that it was unable to eliminate unknown biases, such as dietary habits and family history of diabetes, even after adjusting for numerous covariates. Secondly, it was impossible to consider the impact of fluctuating changes in certain indicators on the study results during the follow-up period, since several indicators, including GGT and HDL-C, were only obtained at the baseline examination. Thirdly, this study only included the Japanese population. However, the risk scores of diabetes developed based on specific ethnic group may not be applicable to other ethnic groups, and therefore the conclusions obtained may be of limited value to non-Japanese populations (40). Fourth, the oral glucose tolerance test (OGTT) test was not considered by the original investigators as a routine item for the diagnosis of T2DM, which could have contributed to the low incidence of diabetes even after a follow-up period of more than 5 years.

## Conclusion

Compared with single GGT and HDL-C, GGT/HDL-C ratio is a more valuable predictor of T2DM, and the risk of T2DM increases in the condition of higher GGT/HDL-C ratio. For clinical application, doctors should provide timely guidance to the medical prevention of T2DM in the future for those with GGT/HDL-C ratios above 17.92 particularly.

## Data availability statement

Publicly available datasets were analyzed in this study. This data can be found here: Okamura, Takuro et al. (2019), Data from: Ectopic fat obesity presents the greatest risk for incident type 2 diabetes: a population-based longitudinal study, Dryad, Dataset, https://doi.org/10.5061/dryad.8q0p192.

## Ethics statement

The studies involving human participants were reviewed and approved by Ethics Committee of the Murakami Memorial Hospital. The patients/participants provided their written informed consent to participate in this study.

## Author contributions

XWC, LB and TYS analyzed the data and drafted the manuscript. XWC collected and assembled the data. SZS and YTS contributed to design and critically revised the manuscript for important intellectual content. All authors read and approved the final version of the manuscript.

## Acknowledgments

We appreciate Okamura et al. for sharing their scientific data.

## Conflict of interest

The authors declare that the research was conducted in the absence of any commercial or financial relationships that could be construed as a potential conflict of interest.

## Publisher’s note

All claims expressed in this article are solely those of the authors and do not necessarily represent those of their affiliated organizations, or those of the publisher, the editors and the reviewers. Any product that may be evaluated in this article, or claim that may be made by its manufacturer, is not guaranteed or endorsed by the publisher.

## Abbreviations

ALT, Alanine aminotransferase; AST, Aspartate transaminase; AUC, Area under the curve; BMI, Body mass index; CI, Confidence interval; DBP, Diastolic blood pressure; DNL, *De novo* lipogenesis; FPG, Fasting plasma glucose; GGT, Gamma-glutamyl transferase; HbA1c, Glycated hemoglobin; HDL-C, High-density lipoprotein cholesterol; HR, Hazard ratio; NAFLD, Non-alcoholic fatty liver disease; OGTT, Oral glucose tolerance test; RCS, Restricted cubic spline; ROC, Receiver-operating-characteristic; SBP, Systolic blood pressure; T1DM, Type 1 diabetes mellitus; T2DM, Type 2 diabetes mellitus.

## References

[1] Bommer C Heesemann E Sagalova V Manne-Goehler J Atun R Bärnighausen T . The global economic burden of diabetes in adults aged 20-79 years: A cost-of-illness study. Lancet Diabetes Endocrinol (2017) 5:423–30. doi: 10.1016/S2213-8587(17)30097-9 28456416

[2] International Diabetes Federation . IDF diabetes atlas (2022). Available at: https://diabetesatlas.org/ (Accessed August 8, 2022).

[3] Zheng Y Ley SH Hu FB . Global aetiology and epidemiology of type 2 diabetes mellitus and its complications. Nat Rev Endocrinol (2018) 14:88–98. doi: 10.1038/nrendo.2017.151 29219149

[4] Petersen MC Shulman GI . Mechanisms of insulin action and insulin resistance. Physiol Rev (2018) 98:2133–223. doi: 10.1152/physrev.00063.2017 PMC6170977 30067154

[5] De Silva NMG Borges MC Hingorani AD Engmann J Shah T Zhang X . Liver function and risk of type 2 diabetes: Bidirectional mendelian randomization study. Diabetes (2019) 68:1681–91. doi: 10.2337/db18-1048 PMC7011195 31088856

[6] Shin JY Chang SJ Shin YG Seo KS Chung CH . Elevated serum gamma-glutamyltransferase levels are independently associated with insulin resistance in non-diabetic subjects. Diabetes Res Clin Pract (2009) 84:152–7. doi: 10.1016/j.diabres.2009.02.004 19264371

[7] Zhao W Tong J Liu J Liu J Li J Cao Y . The dose-response relationship between gamma-glutamyl transferase and risk of diabetes mellitus using publicly available data: A longitudinal study in Japan. Int J Endocrinol (2020) 2020:5356498. doi: 10.1155/2020/5356498 32215009 PMC7054786

[8] Lee YS Cho Y Burgess S Davey Smith G Relton CL Shin S-Y . Serum gamma-glutamyl transferase and risk of type 2 diabetes in the general Korean population: A mendelian randomization study. Hum Mol Genet (2016) 25:3877–86. doi: 10.1093/hmg/ddw226 27466193

[9] Fiorentino TV Succurro E Marini MA Pedace E Andreozzi F Perticone M . HDL cholesterol is an independent predictor of β-cell function decline and incident type 2 diabetes: A longitudinal study. Diabetes Metab Res Rev (2020) 36:e3289. doi: 10.1002/dmrr.3289 31922637

[10] Cao C Hu H Zheng X Zhang X Wang Y He Y . Non-linear relationship between high-density lipoprotein cholesterol and incident diabetes mellitus: A secondary retrospective analysis based on a Japanese cohort study. BMC Endocr Disord (2022) 22:163. doi: 10.1186/s12902-022-01074-8 35717187 PMC9206738

[11] Feng G Feng L Zhao Y . Association between ratio of γ-glutamyl transpeptidase to high-density lipoprotein cholesterol and prevalence of nonalcoholic fatty liver disease and metabolic syndrome: A cross-sectional study. Ann Transl Med (2020) 8:634. doi: 10.21037/atm-19-4516 32566571 PMC7290624

[12] Okamura T Hashimoto Y Hamaguchi M Ohobra A Kojima T Fukui M . Ectopic fat obesity presents the greatest risk for incident type 2 diabetes: A population-based longitudinal study. Dryad (2019). doi: 10.5061/dryad.8q0p192 29717276

[13] Okamaru T Hashimoto Y Hamaguchi M Obara A Kojima T Fukui M Ectopic fat obesity presents the greatest risk for incident type 2 diabetes: A population-based longitudinal study. Int J Obes (Lond) (2019) 43, 139–48. 29717276 10.1038/s41366-018-0076-3

[14] Shang L Li R Zhao Y Sun H Tang B Hou Y . Association between Chinese visceral adiposity index and incident type 2 diabetes mellitus in Japanese adults. Diabetes Metab Syndr Obes (2021) 14:3743–51. doi: 10.2147/DMSO.S322935 PMC8402978 34466009

[15] Guasch-Ferré M Hruby A Toledo E Clish CB Martínez-González MA Salas-Salvadó J . Metabolomics in prediabetes and diabetes: A systematic review and meta-analysis. Diabetes Care (2016) 39:833–46. doi: 10.2337/dc15-2251 PMC4839172 27208380

[16] Gao F Huang XL Jiang XP Xue M Li YL Lin XR . Independent effect of alanine transaminase on the incidence of type 2 diabetes mellitus, stratified by age and gender: A secondary analysis based on a large cohort study in China. Clin Chim Acta (2019) 495:54–9. doi: 10.1016/j.cca.2019.03.1636 30946812

[17] Schneider AL Lazo M Ndumele CE Pankow JS Coresh J Clark JM . Liver enzymes, race, gender and diabetes risk: The atherosclerosis risk in communities (ARIC) study. Diabetes Med (2013) 30:926–33. doi: 10.1111/dme.12187 PMC3715563 23510198

[18] Cherrington AD Edgerton D Sindelar DK . The direct and indirect effects of insulin on hepatic glucose production in vivo. Diabetologia (1998) 41:987–96. doi: 10.1007/s001250051021 9754815

[19] Leavens KF Birnbaum MJ . Insulin signaling to hepatic lipid metabolism in health and disease. Crit Rev Biochem Mol Biol (2011) 46:200–15. doi: 10.3109/10409238.2011.562481 21599535

[20] Brown MS Goldstein JL . Selective versus total insulin resistance: A pathogenic paradox. Cell Metab (2008) 7:95–6. doi: 10.1016/j.cmet.2007.12.009 18249166

[21] Wu X Chen K Williams KJ . The role of pathway-selective insulin resistance and responsiveness in diabetic dyslipoproteinemia. Curr Opin Lipidol (2012) 23:334–44. doi: 10.1097/MOL.0b013e3283544424 22617754

[22] Smith BW Adams LA . Nonalcoholic fatty liver disease and diabetes mellitus: pathogenesis and treatment. Nat Rev Endocrinol (2011) 7:456–65. doi: 10.1038/nrendo.2011.72 21556019

[23] Hua S Qi Q Kizer JR Williams-Nguyen J Strickler HD Thyagarajan B . Association of liver enzymes with incident diabetes in US Hispanic/Latino adults. Diabetes Med (2021) 8:e14522. doi: 10.1111/dme.14522 33434318

[24] Park JY Han K Kim HS Cho JH Yoon KH Kim MK . Cumulative exposure to high γ-glutamyl transferase level and risk of diabetes: A nationwide population-based study. Endocrinol Metab (Seoul) (2022) 37:272–80. doi: 10.3803/EnM.2022.1416 PMC9081297 35413781

[25] Wang H Li L Zhang S . Non-linear relationship between gamma-glutamyl transferase and type 2 diabetes mellitus risk: Secondary analysis of a prospective cohort study. J Int Med Res (2020) 48:300060520937911. doi: 10.1177/0300060520937911 32662704 PMC7361500

[26] Rehman K Akash MSH . Mechanism of generation of oxidative stress and pathophysiology of type 2 diabetes mellitus: How are they interlinked? J Cell Biochem (2017) 118:3577–85. doi: 10.1002/jcb.26097 28460155

[27] Gohel MG Chacko AN . Serum GGT activity and hsCRP level in patients with type 2 diabetes mellitus with good and poor glycemic control: An evidence linking oxidative stress, inflammation and glycemic control. J Diabetes Metab Disord (2013) 12:56. doi: 10.1186/2251-6581-12-56 24360326 PMC7962520

[28] Thorand B Löwel H Schneider A Kolb H Meisinger C Fröhlich M . C-reactive protein as a predictor for incident diabetes mellitus among middle-aged men: results from the MONICA augsburg cohort study, 1984-1998. Arch Intern Med (2003) 163:93–9. doi: 10.1001/archinte.163.1.93 12523922

[29] Anderson ME Allison RD Meister A . Interconversion of leukotrienes catalyzed by purified gamma-glutamyl transpeptidase: Concomitant formation of leukotriene D4 and gamma-glutamyl amino acids. Proc Natl Acad Sci U.S.A. (1982) 79:1088–91. doi: 10.1073/pnas.79.4.1088 PMC345905 6122208

[30] Rohatgi A Westerterp M von Eckardstein A Remaley A Rye KA . HDL in the 21st century: A multifunctional roadmap for future HDL research. Circulation (2021) 143:2293–309. doi: 10.1161/CIRCULATIONAHA.120.044221 PMC8189312 34097448

[31] Hirano M Nakanishi S Kubota M Maeda S Yoneda M Yamane K . Low high-density lipoprotein cholesterol level is a significant risk factor for development of type 2 diabetes: Data from the Hawaii-Los Angeles-Hiroshima study. J Diabetes Investig (2014) 5:501–6. doi: 10.1111/jdi.12170 PMC4188106 25411616

[32] Abbasi A Corpeleijn E Gansevoort RT Gans RO Hillege HL Stolk RP . Role of HDL cholesterol and estimates of HDL particle composition in future development of type 2 diabetes in the general population: The PREVEND study. J Clin Endocrinol Metab (2013) 98:E1352–9. doi: 10.1210/jc.2013-1680 23690306

[33] Yuan S Larsson SC . An atlas on risk factors for type 2 diabetes: A wide-angled mendelian randomisation study. Diabetologia (2020) 63:2359–71. doi: 10.1007/s00125-020-05253-x PMC7527357 32895727

[34] Cao X Tang Z Zhang J Li H Singh M Sun F . Association between high-density lipoprotein cholesterol and type 2 diabetes mellitus among Chinese: The Beijing longitudinal study of aging. Lipids Health Dis (2021) 20:71. doi: 10.1186/s12944-021-01499-5 34273996 PMC8286580

[35] Drew BG Duffy SJ Formosa MF Natoli AK Henstridge DC Penfold SA . High-density lipoprotein modulates glucose metabolism in patients with type 2 diabetes mellitus. Circulation (2009) 119:2103–11. doi: 10.1161/CIRCULATIONAHA.108.843219 19349317

[36] Swerdlow DI Preiss D Kuchenbaecker KB Holmes MV Engmann JE Shah T . HMG-coenzyme a reductase inhibition, type 2 diabetes, and bodyweight: Evidence from genetic analysis and randomised trials. Lancet (2015) 385:351–61. doi: 10.1016/S0140-6736(14)61183-1 PMC4322187 25262344

[37] Xepapadaki E Nikdima I Sagiadinou EC Zvintzou E Kypreos KE . HDL and type 2 diabetes: The chicken or the egg? Diabetologia (2021) 64:1917–26. doi: 10.1007/s00125-021-05509-0 34255113

[38] Tabara Y Arai H Hirao Y Takahashi Y Setoh K Kawaguchi T . Different inverse association of large high-density lipoprotein subclasses with exacerbation of insulin resistance and incidence of type 2 diabetes: The nagahama study. Diabetes Res Clin Pract (2017) 127:123–31. doi: 10.1016/j.diabres.2017.03.018 28365559

[39] Liu J van Klinken JB Semiz S van Dijk KW Verhoeven A Hankemeier T . A mendelian randomization study of metabolite profiles, fasting glucose, and type 2 diabetes. Diabetes (2017) 66:2915–26. doi: 10.2337/db17-0199 28847883

[40] Ha KH Lee YH Song SO Lee JW Kim DW Cho KH . Development and validation of the Korean diabetes risk score: A 10-year national cohort study. Diabetes Metab J (2018) 42:402–14. doi: 10.4093/dmj.2018.0014 PMC6202558 30113144

